# Myocardial fibrosis by late gadolinium enhancement cardiovascular magnetic resonance in myotonic muscular dystrophy type 1: highly prevalent but not associated with surface conduction abnormality

**DOI:** 10.1186/s12968-019-0535-6

**Published:** 2019-05-02

**Authors:** Andrea Cardona, William D. Arnold, John T. Kissel, Subha V. Raman, Karolina M. Zareba

**Affiliations:** 1 Division of Cardiovascular Medicine, The Ohio State University Wexner Medical Center, 473 W 12th Ave, Suite 200, Columbus, OH 43210 USA; 2 Division of Cardiology, University of Perugia, Rome, Italy; 3 Division of Neuromuscular Disorders, The Ohio State University Wexner Medical Center, Columbus, OH USA

**Keywords:** Myotonic muscular dystrophy, Cardiovascular magnetic resonance, Myocardial fibrosis, Electrocardiogram, Pacemaker, Late gadolinium enhancement

## Abstract

**Background:**

Conduction disease and arrhythmias represent a major cause of mortality in myotonic muscular dystrophy type 1 (MMD1). Permanent pacemaker (PPM) implantation is the cornerstone of therapy to reduce cardiovascular mortality in MMD1. Cardiovascular magnetic resonance (CMR) studies demonstrate a high prevalence of myocardial fibrosis in MMD1, however the association between CMR myocardial fibrosis with late gadolinium enhancement (CMR-LGE) and surface conduction abnormality is not well established in MMD1.

We investigated whether myocardial fibrosis by CMR-LGE is associated with surface conduction abnormalities meeting criteria for PPM implantation according to current guidelines in a cohort of patients with genetically confirmed MMD1.

**Methods:**

Patients with genetically confirmed MMD1 were retrospectively evaluated. 12-lead electrocardiography (ECG) performed within 6 months of CMR was necessary for inclusion. The severity and extent of MMD1 was quantified using a validated Muscular Impairment Rating Scale (MIRS). Based on current guidelines for device-based therapy of cardiac rhythm abnormalities, we defined surface conduction abnormality as the presence of ECG alterations meeting criteria for PPM implant (class I or II indications): PR interval > 200 ms (type I atrioventricular (AV) block) and/or mono or bifascicular block (QRS > 120 ms), or evidence of advanced AV block. Balanced steady-state free precession sequences (bSSFP) were used for assessment of left ventricular (LV) volumes and ejection fraction. MOdified Look-Locker Inversion Recovery (MOLLI) acquisition schemes were used to acquire T1 maps. Patients’ charts were reviewed up to 12 months post-CMR for occurrence of PPM implantation.

**Results:**

Fifty-two patients (38% male, 41 ± 14 years) were included. Overall, 31 (60%) patients had a surface conduction abnormality and 22 (42%) demonstrated midwall myocardial fibrosis by CMR-LGE. After a median of 57 days from CMR exam, 15 patients (29%) underwent PPM implantation. Subjects with vs. without surface conduction abnormality had significantly longer disease length (15.5 vs. 7.8 years, *p* = 0.015) and higher disease severity on the MIRS scale (*p* = 0.041). High prevalence of myocardial fibrosis by CMR-LGE was detected in subjects with and without surface conduction abnormality with no significant difference between the two cohorts (42% vs. 43%, *p* = 0.999). By multivariate logistic regression analysis, disease length was the only independent variable associated with surface conduction abnormality (OR 1.071, 95%CI 1.003–1.144, *p* = 0.040); while CMR-LGE was not associated with conduction abnormality (ρ = − 0.009, *p* = 0.949).

**Conclusions:**

Myocardial fibrosis by CMR-LGE is highly prevalent in MMD1 but not related to surface conduction abnormality meeting current guideline criteria for PPM implantation .

**Electronic supplementary material:**

The online version of this article (10.1186/s12968-019-0535-6) contains supplementary material, which is available to authorized users.

## Introduction

Myotonic muscular dystrophy type 1 (MMD1) is the most common muscular dystrophy in adults and is characterized by progressive muscle degeneration leading to disabling weakness and wasting with myotonia, in combination with multisystem involvement [1]. MMD1 is characterized by an autosomal dominant inheritance [2]. CTG repeat expansion in the myotonic dystrophy protein kinase gene (DMPK) is the mutation underlying this condition.

Conduction abnormalities and cardiac arrhythmias represent a major cause of mortality in MMD1; thus the prevention of sudden cardiac death (SCD) is central to patient management [3–6]. Cardiac involvement initially manifests as asymptomatic electrocardiographic (ECG) abnormalities, typically prolongation of the PR and QRS intervals progressing to more advanced conduction disease including sinus node dysfunction and heart block as well as atrial tachycardia, and ventricular tachycardia or fibrillation [3–5]. Progression of conduction system disease to complete atrioventricular (AV) block is the presumed cause of SCD in a high proportion of patients [3–5]. Implantation of a permanent pacemaker (PPM) has been found useful even in asymptomatic patients with an abnormal resting ECG or with HV interval prolongation during electrophysiological study as the disease course can have unpredictable progression to advanced conduction disease [5, 6]. Therefore, permanent pacing has been recommended by the American College of Cardiology and the American Heart Association when complete AV block or advanced high-degree AV block are detected (class I indication), or prophylactically for patients presenting with first-degree AV or fascicular block on the ECG (class IIb indication) [5].

Myocardial fibrosis has been identified in autopsies from patients with MMD1 together with fatty infiltration and myocyte hypertrophy and degeneration [7, 8]. Cardiac fibrosis and fatty infiltration most commonly affect the myocardium and the His–Purkinje system, but may also involve the sino-atrial and AV nodes thus providing a substrate for conduction abnormalities and arrhythmias [7, 9–13]. Cardiovascular magnetic resonance (CMR) studies using late gadolinium enhancement imaging (CMR-LGE), where abnormal appearance of the myocardium often represents fibrosis, have revealed a prevalence of myocardial fibrosis by LGE in MMD ranging from 0 to 40% [9–14]. However, variable associations between conduction abnormalities and myocardial fibrosis by CMR-LGE have been reported [9–13]. To date, there are no studies that have specifically explored the relationship between myocardial fibrosis by CMR-LGE and surface conduction abnormalities meeting criteria for PPM implant in MMD1, a procedure that is advocated for SCD prevention in patients with this disease [4, 5].

We sought to evaluate the association between the presence and extent of CMR-LGE myocardial fibrosis and surface conduction abnormalities meeting criteria for PPM implantation according to current guidelines in a cohort of patients with genetically confirmed MMD1.

## Methods

### Population selection

We retrospectively evaluated consecutive patients from the Muscular Dystrophy Clinic of The Ohio State University Medical Center, with genetically confirmed diagnosis of MMD1 referred for a clinical CMR between April 2012 and March 2017. Subsequent decision-making regarding PPM implantation was done at the clinical discretion of the referring provider, typically based on ECG findings, and did not incorporate CMR data. Standard 12-lead ECG performed within 6 months of CMR exam was necessary for inclusion. Exclusion criteria included standard contraindications to perform a CMR exam: severe claustrophobia, presence of a pacing device, ferromagnetic foreign body, active implant, known allergy to gadolinium-based contrast, or advanced kidney disease (e.g. glomerular filtration rate < 30 mL/min/1.73 m2). The Ohio State University Institutional Review Board approved this retrospective study and waived informed consent.

### Clinical assessment

All patients underwent a comprehensive clinical evaluation and physical examination during their clinical visit. The severity and extent of MMD was quantified using a validated Muscular Impairment Rating Scale (MIRS) related to the clinical distribution and extent of muscle weakness and myotonia [15]. The New York Heart Association (NYHA) classification system was used to assess exercise capacity. Patient’s charts were reviewed up to 12 months after CMR to record date of PPM implant or occurrence of any other major adverse cardiac events defined as heart failure, ventricular arrhythmias, or death.

### Electrophysiologic analysis

Electrical parameters were assessed from a standard 12-lead surface ECG. Based on current American College of Cardiology Foundation/ American Heart Association/Heart Rhythm Society (ACCF/AHA/HRS) guidelines for device-based therapy of cardiac rhythm abnormalities [5], we defined surface conduction abnormality as the presence of ECG alterations meeting criteria for PPM implant (class I or II indications) in subjects with MMD. Accordingly, surface conduction abnormality was defined as evidence of PR interval > 200 ms (type I AV block) and/or mono or bifascicular block (QRS > 120 ms), or evidence of an advanced AV block [5]. Left ventricular (LV) hypertrophy (LVH) was assessed by Cornell criteria [16]. According to current consensus documents for Standardization and Interpretation of the Electrocardiogram from the ACCF/AHA/HRS, standard criteria were adopted to define the presence of left anterior fascicular block (LAFB), left posterior fascicular block (LPFB), left bundle branch block (LBBB), right bundle branch block (RBBB), and non-specific intraventricular conduction delay (IVCD) [17]. The QT interval was obtained via automated ECG analysis in leads II, V2, and V5. The QTc interval was calculated using the Bazett formula. Electrophysiological (EP) study data and pacing device information were retrieved from patients’ charts. Referrals for intracardiac EP studies were performed in the presence of symptomatic or asymptomatic AV conduction abnormalities (first-degree or higher AV block) with or without QRS interval ≥ 120 ms, and/or presence of palpitations, syncope, near syncope or documented arrhythmias.

### Cardiovascular magnetic resonance protocol

All images were acquired on a single 1.5T CMR scanner with a 12-element phased array chest coil (MAGNETOM Avanto, Siemens Healthineers, Erlangen, Germany). Balanced steady-state free precession sequences (bSSFP) were used for assessment of LV volumes, ejection fraction (LVEF) and LV mass. LGE imaging was performed using a gradient-echo inversion recovery sequence with magnitude and phase sensitive inversion recovery reconstructions 10 min after 0.2 mmol/kg of gadolinium based contrast agent [18]. LV volumes and LVEF were measured from contiguous LV short-axis cine images using semi-automated software for endocardial segmentation using endocardial and epicardial contours at end-systole and end-diastole with Simpson’s rule. LV mass was calculated from the total end-diastolic myocardial volume multiplied by the specific gravity of the myocardium (1.05 g/ml) [19]. Presence of LGE was assessed by 2 expert operators blinded to clinical data (AC, KZ), with a third providing adjudication if necessary (SVR). The LGE (grams) mass was quantified by a blinded operator (AC) using a dedicated software implementing the full-width at half-maximum technique (CMR42, Circle Cardiovascular Imaging Inc. Calgary, Alberta, Canada) and indexed as a percentage of LV mass [20]. MOdified Look-Locker Inversion Recovery (MOLLI) acquisition schemes were used to acquire T1 maps produced using vendor software before and 15 min after administration of contrast. The MOLLI 3(3)3(3)5 sequence was used in the first 23 patients and subsequently the MOLLI 5(3)3 sequence was used in 29 patients. T1 values and extracellular volume fraction (ECV) were measured and calculated utilizing interventricular septal values from the mid short axis view. The region of interest was placed in the mid myocardium with manual tracing to avoid partial volume effects and regions of LGE if present [21, 22]. Myocardial ECV was calculated as previously described [23]. Reference values for normal pre- and post-contrast myocardial T1 values and ECV for the MOLLI 3(3)3(3)5 sequence are based on data of 18 healthy subjects at our institution (45 ± 18 years, 39% female), and are as follows: native T1 940 ± 28 ms, post-contrast T1 403 ± 42 ms, and ECV 24.3 ± 2.3%. Reference values for the myocardium utilizing the MOLLI 5(3)3 sequence based on 44 healthy subjects at our institution (36 ± 15 years, 59% female) are as follows: native T1 999 ± 31 ms, post-contrast T1 453 ± 30 ms, and ECV 23.8 ± 2.6%. In line with previous literature, the abnormal value for ECV was 29%, corresponding to 2SD above the reference value [24].

### Statistical analysis

Data are presented as mean ± standard deviation (SD) or as median and interquartile range (IQR) for continuous variables and as proportions for categorical variables. The mean values of continuous, normally distributed variables were compared with the 2-sample t-test. Comparison of median values or proportions was done with the 2-sample Wilcoxon rank-sum test, and Fisher exact test, respectively. Univariate logistic regression analysis was used to find individual variables associated with surface conduction abnormality. After testing for collinearity, multivariate stepwise-backward logistic regression analysis was used to find independent variables associated with surface conduction abnormality. The association between CMR-LGE, surface conduction abnormality and PPM implant was also assessed with Spearman correlation analysis. An additional analysis was performed to compare clinical and ECG characteristics according to the presence vs. absence of LGE. Statistical significance was set at two tailed *p* <  0.05. SPSS Statistic 21.0 (Statistical Package for the Social Sciences (SSPS) International Business Machines, Inc., Armonk, New York, USA) was used for all statistical analyses.

## Results

### Patients characteristics

Fifty-two patients with genetically confirmed MMD1 (median CTG repeats = 500, IQR = 200–1163), 38% male, 41 ± 14 years, met criteria for inclusion in the study. Overall, thirty-one patients (60%) demonstrated surface conduction abnormality: 20 (38%) with a prolonged PR interval (mean 232 ± 51 ms); 5 (9.6%) with a prolonged PR interval associated with RBBB; 2 (3.8%) patients had a prolonged PR interval associated with LBBB; 2 (3.8%) had LBBB alone, one patient had RBBB alone, and one had Mobitz AV block type I. Baseline demographic, clinical and ECG characteristics according to presence or absence of surface conduction abnormality are presented in Table 1.

**Table 1:** Demographic, clinical and electrocardiographic findings in the study cohort

	Whole Cohort (*N* = 52)	Conduction Abnormality Positive (*N* = 31)	Conduction Abnormality Negative (*N* = 21)	*P* value
Demographic data				
Age, years	41.2 ± 13.9	43.7 ± 11.9	37.4 ± 15.9	0.129
Male gender, N (%)	20 (38)	11 (35)	9 (43)	0.772
BMI, kg/m^2^	25.5 ± 5.8	25.8 ± 6.2	24.9 ± 5.1	0.618
DM, N (%)	5 (10)	4 (13)	1 (5)	0.637
HTN, N (%)	3 (6)	1 (3)	2 (9)	0.558
Smoking, N (%)	2 (4)	2 (6)	0 (0)	0.494
HLP, N (%)	5 (10)	4 (13)	1 (5)	0.637
History of CAD, N (%)	1 (2)	1 (3)	0 (0)	0.999
BB, N (%)	4 (8)	3 (10)	1 (5)	0.639
ACE-i, N (%)	3 (6)	2 (6)	1 (5)	0.999
ARB, N (%)	1 (2)	1 (3)	0 (0)	0.999
MCRA, N (%)	4 (8)	4 (13)	0 (0)	0.138
Statin, N (%)	6 (11)	4 (13)	2 (9)	0.999
Myotonic Muscular Dystrophy Characteristics				
Age of onset, years	28.8 ± 15.5	28.2 ± 17.1	29.6 ± 13.1	0.749
Disease length, years	12.4 ± 12.7	15.5 ± 14.8	7.8 ± 6.9	0.015*
CTG Repeats	500 (200–1163)	280 (142–772)	575 (450–1450)	0.137
MIRS scale	3 (3–4)	3 (3–4)	3 (2–3)	0.041*
Clinical data				
SBP, mmHg	126 ± 18	125 ± 18	126 ± 19	0.798
DBP, mmHg	74 ± 10	74 ± 11	75 ± 8	0.733
HR, bpm	74 ± 14	74 ± 16	75 ± 12	0.793
NYHA class	2 (1–2)	2 (1–2)	1 (1–2)	0.900
Hematocrit, %	42.1 ± 3.8	42.3 ± 3.4	41.7 ± 4.4	0.592
Electrocardiographic Data				
PR, ms	199 ± 47	223 ± 48	165 ± 16	< 0.001
QRS, ms	103 ± 20	111 ± 22	94 ± 11	0.001
QT, ms	404 ± 42	412 ± 47	392 ± 31	0.070
QTc, ms	428 ± 32	431 ± 38	423 ± 23	0.359
Frontal QRS-T angle	41 ± 41	45 ± 44	36 ± 36	0.447
LVH-Cornell, N (%)	13 (25)	11 (35)	2 (9)	0.050

Data are presented as mean ± SD, N (%), or median (interquartile range). *p < 0.05 considered significant. *BMI* body mass index, *DM* diabetes mellitus, *HLP* hyperlipidemia, *CAD* coronary artery disease, *BB* beta-blockers, *ACE-i* Angiotensin-converting enzyme inhibitors, *ARB* Angiotensin receptor blockers, *MCRA* aldosterone antagonists, *MIRS* Muscular Impairment Rating Scale, *SBP* systolic blood pressure, *DBP* diastolic blood pressure, *HR* heart rate, *NYHA* New York Heart Association class, *LVH* left ventricular hypertrophy

Subjects with conduction abnormality tended to be older, had a longer disease length (15.5 vs. 7.8 years, *p* = 0.015) and demonstrated higher MIRS scores (*p* = 0.041) compared to subjects without conduction abnormalities (Table 1). As expected they also demonstrated a longer PR and QRS intervals as compared to subjects without conduction abnormality. Alternatively, no differences were found between the two groups in the prevalence of major cardiovascular risk factors or medications, CTG repeats, or functional status (Table 1).

After a median time of 57 days from the CMR exam, 15 patients (29%) received a PPM: 7 subjects were implanted due to coexistence of first degree AV and fascicular block, 4 for presence of bifascicular block associated with either a first degree AV block or a prolonged HV interval, 2 for a prolonged HV interval, one for presence of second degree AV block (Mobitz type I), and one for presence of I degree AV block (Table 2). No cardiac death or major cardiovascular events occurred during the 12 months of observation.

**Table 2:** Characteristics of Subjects with MMD undergoing PPM implant

#	Age (years)	Sex	Disease Length (years)	NYHA Class	MIRS Scale	LVEDVI, (ml/m^2^)	LVESVI, (ml/m^2^)	LVEF, (%)	LGE Any	LGE (%)	ECV (%)	Rhythm	HR (bpm)	PR (ms)	QRS (ms)	QTc	Surface Conduction Disease	AV block type	LAFB	LBBB	RBBB	IVCD	Bif. Block	HV (ms)	HV > 70 ms
1	48	F	28	3	2	65.8	29.5	55.2	0	0	27.5	sinus	62	220	186	519	1	I	0	1	0	0	0	73	1
2	36	M	28	1	5	87.0	38.5	55.7	0	0	22.4	sinus	54	236	116	402	1	I	0	0	0	1	0	77	1
3	44	M	5	1	3	78.6	36.2	53.9	1	2.7	26.2	Mobitz 1	35	440	102	366	1	II	0	0	0	0	0	N/A	N/A
4	31	F	9	2	4	73.3	29.3	60.0	0	0	26.8	sinus	103	220	86	466	1	I	1	0	0	0	0	N/A	N/A
5	42	F	5	1	4	63.9	32.2	49.6	0	0	33.8	sinus	74	220	116	477	1	I	1	0	0	1	0	86	1
6	34	F	12	1	3	61.1	35.3	42.2	1	5.4	27.2	sinus	70	210	112	481	1	I	0	0	0	1	0	N/A	N/A
7	61	F	12	2	5	46.8	20.0	57.3	0	0	21.7	sinus	74	220	118	463	1	I	0	0	0	1	0	N/A	N/A
8	53	M	36	1	3	50.0	15.6	68.8	1	13.7	21.2	sinus	54	250	132	411	1	I	0	1	0	0	0	95	1
9	55	M	1	1	3	43.5	12.0	72.4	1	16.2	24.3	sinus	61	220	142	446	1	I	0	0	1	0	0	71	1
10	48	F	30	2	3	76.3	26.8	64.8	0	0	31.8	sinus	49	236	120	419	1	I	1	0	0	1	1	N/A	N/A
11	40	F	1	2	4	61.0	28.8	53.3	1	11.8	25.4	sinus	80	156	132	452	1	None	1	0	0	0	0	75	1
12	43	F	14	1	3	73.2	27.5	62.5	0	0	29.9	sinus	68	150	142	472	1	None	1	0	1	0	1	80	1
13	33	M	8	2	3	70.5	32.4	54.0	0	0	25.0	sinus	68	240	84	421	1	I	0	0	0	0	0	N/A	N/A
14	31	F	26	2	3	64.8	27.2	58.1	0	0	23.4	sinus	83	205	128	434	1	I	1	0	1	0	1	74	1
15	26	M	18	2	3	57.7	24.4	57.7	1	5.1	20.7	sinus	98	205	96	439	1	I	1	0	1	0	1	72	1

*LVEDVI* left ventricular end-diastolic volume index, *LVESVI* left ventricular end-systolic volume index, *LVEF* left ventricular ejection fraction, *LGE* late gadolinium enhancement, *ECV* extracellular volume fraction, *HR* heart rate, *LAFB* left anterior fascicular block, *LBBB* left bundle branch block, *RBBB* right bundle branch block, *IVCD* nonspecific intraventricular conduction delay, *Bif*. *block* bifascicular block, *HV His*-Ventricular interval

### CMR findings

Overall, twenty-two subjects demonstrated midwall myocardial fibrosis by CMR-LGE, corresponding to a prevalence of 42%. The LVEF, and LV volumes were within normal range for the entire population (LVEF 60 ± 6%, LV end-diastolic volume index 65 ± 15 ml/m^2^, LV end-systolic volume index 26 ± 8 ml/m^2^) (Table 3).

**Table 3:** CMR Findings

CMR data	Whole Cohort (N = 52)	Conduction Abnormality Positive (N = 31)	Conduction Abnormality Negative (N = 21)	P value
LVEF (%)	60 ± 6	59 ± 6	60 ± 6	0.687
LV EDVI, ml/m^2^	65 ± 15	65 ± 14	66 ± 15	0.728
LV ESVI, ml/m^2^	26 ± 8	26 ± 8	26 ± 7	0.940
LV mass index, g/m^2^	44 ± 11	45 ± 14	43 ± 6	0.563
LAVI, ml/m^2^	30 ± 11	31 ± 10	29 ± 12	0.634
LGE, N (%)	22 (42%)	13 (42)	9 (43)	0.999
LGE mass, g	2.4 ± 3.6	2.4 ± 3.8	2.3 ± 3.4	0.876
LGE, %	3.1 ± 4.6	3.0 ± 4.6	3.1 ± 4.7	0.975
ECV, %	25 ± 3	26 ± 3	24 ± 3	0.050

Data are presented as mean ± SD or N (%). *LVEF* left ventricular ejection fraction, *EDVI* end-diastolic volume index, *ESVI* end-systolic volume index, *LAVI* left atrial volume index, *LGE* late gadolinium enhancement, *ECV* extracellular volume

The median time between the CMR exam and the ECG was 21 days (IQR 7–54). Subjects with conduction abnormality demonstrated similar LVEF, LV volumes, LV mass, and left atrial (LA) volumes compared to those without conduction abnormality (Table 3). Importantly, high prevalence of myocardial fibrosis by CMR-LGE was detected in subjects with and without surface conduction abnormality but no significant difference was noted between the two groups (42% vs. 43%, *p* = 0.999). Further, LGE mass and LGE% were similar between the two groups. ECV tended to be higher in the conduction abnormality group, albeit values were within the normal range in both groups (26 ± 3 vs. 24 ± 3, *p* = 0.050). Data on native myocardial and post-contrast T1 values according to different MOLLI sequences and presence vs. absence of conduction abnormality are presented in Additional file 1: Table S1.

**Fig. 1:**
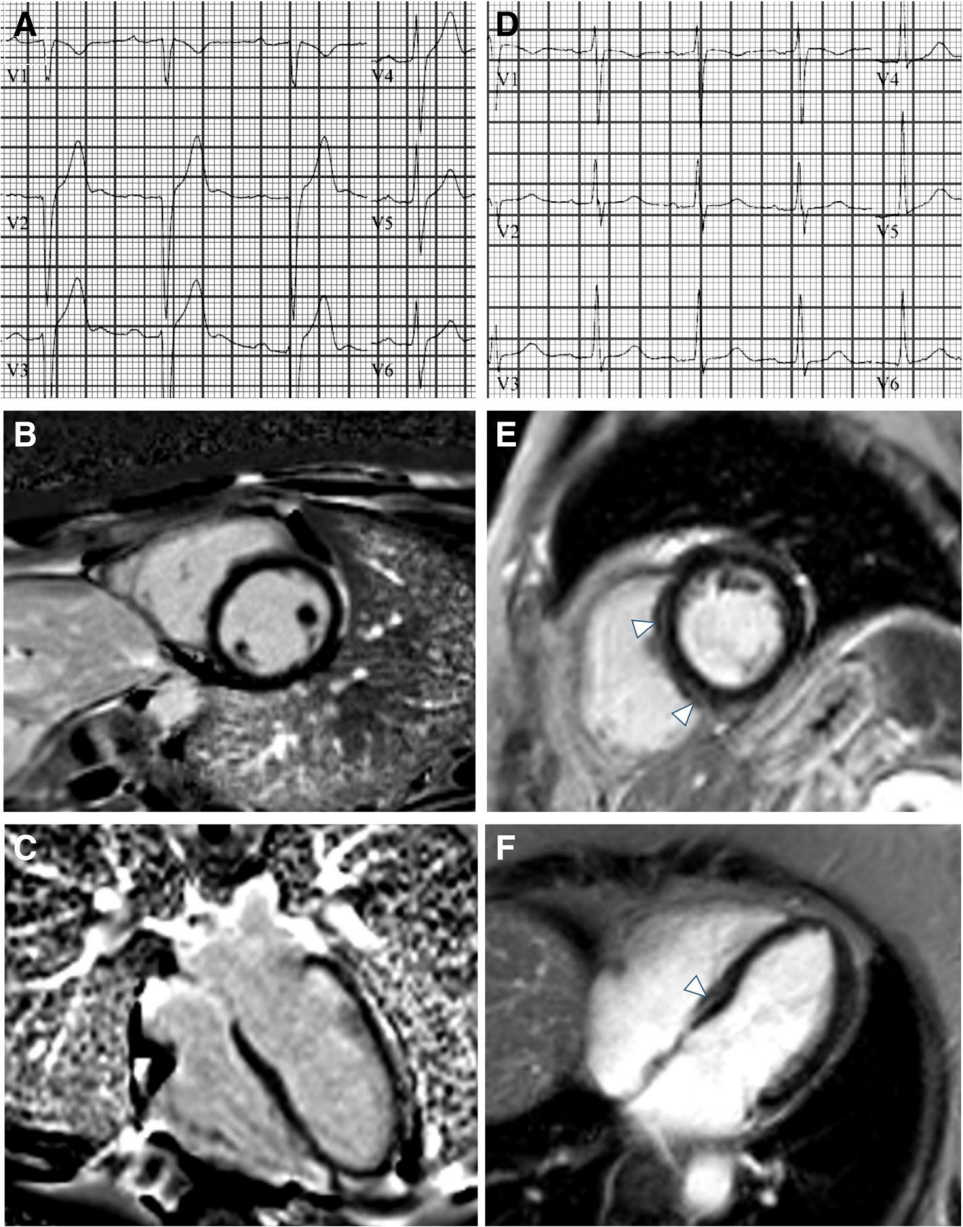
Representative ECG and CMR findings in the study cohort. Panels A shows an abnormal ECG with prolonged PR interval and borderline QRS interval of a subject with no evidence of myocardial fibrosis by CMR-LGE (B, C). Panels D shows a normal ECG of a subjects with evidence of midwall fibrosis mostly evident in the interventricular septum (arrows in E, and F)

Of 22 LGE-positive subjects, all demonstrated midwall fibrosis; in 17 subjects (77%) the interventricular septum was involved, the inferior and inferolateral segments were involved in four subjects (18%), and the anterior wall was involved in one subject (5%). An analysis of clinical and ECG data according to the presence vs. absence of LGE was also conducted (Additional file 1: Tables S2 and S3). There were no significant differences in ECG characteristics in LGE positive vs.LGE negative patients. Fig. 1 demonstrates representative ECG and CMR findings in 2 patients. Additionally, we have provided a Additional file 1: Figure. S1 which includes representative ECG and T1 maps in 2 patients.

### Variables associated with surface conduction abnormality

Disease length and Cornell voltage were associated with surface ECG conduction abnormality on univariate analysis. Disease length was the only independent variable associated with conduction abnormality in the multivariate model (OR 1.071, 95%CI 1.003–1.144, *p* = 0.040; Table 4). Importantly, the presence of myocardial fibrosis by CMR-LGE, LGE mass, or LGE% was not associated with surface conduction abnormality (full regression analysis is shown in Additional file 1: Table S4). Additionally, myocardial fibrosis by CMR-LGE was not associated with surface conduction abnormality by Spearman correlation analysis (ρ = − 0.009, *p* = 0.949) nor was LGE% (ρ = 0.004, *p* = 0.976) or LGE mass (ρ = − 0.022, *p* = 0.878). Similar findings were also true for the association between CMR-LGE and PPM implant (ρ = − 0.03, *p* = 0.834).

**Table 4:** Variables Associated with Surface Conduction Abnormality

Variables	Exp (B) or OR	95% CI	*P* value
Univariate Analysis			
Disease length, years	1.071	1.003–1.144	0.040*
Cornell voltage, mm	1.090	1.007–1.179	0.033*
Presence of CMR-LGE	0.963	0.314–2.953	0.947
Multivariate Analysis^a^			
Disease length, years	1.071	1.003–1.144	0.040*

OR is computed for unit-change for continuous and ordinal predictors. ^a^Multivariate model is based on stepwise-backward conditional regression analysis. *p < 0.05 considered significant. *CMR* cardiac magnetic resonance, *LGE* late gadolinium enhancement

## Discussion

Our study demonstrates that myocardial fibrosis assessed by CMR-LGE is highly prevalent in patients with MMD1 but is not associated with surface conduction abnormality meeting criteria for PPM implant per current guidelines. An important aspect of our study is the high prevalence of myocardial fibrosis by CMR-LGE in subjects without surface conduction abnormality. This relevant finding deserves further attention to better understand the role of CMR in risk stratification of patients with myotonic dystrophy in longer-term follow-up.

In MMD1, conduction system abnormalities can evolve into complete AV block and can be associated with potentially fatal asystole or ventricular arrhythmias that result in SCD [3, 4, 25–27]. However, the cause of a dysfunctional conduction system in MMD is yet to be determined. Myocardial fibrosis can affect the structural integrity and electrical conductive properties of cardiac muscle leading to heart failure and arrhythmias [28, 29]. However, the association between myocardial fibrosis and surface conduction abnormalities in MMD1 has not been well defined and contrasting evidence exists in this regard [10, 11]. In a previous CMR study including 80 patients with MMD1 myocardial fibrosis was associated with concomitant abnormal ECG; however, the prevalence of fibrosis was rather low (13%) [10]. A subsequent study involving 30 subjects with MMD1, showed a higher prevalence of myocardial fibrosis (40%) and no association with abnormal ECG findings [9]. Our findings confirm a high prevalence of myocardial fibrosis (42%) with no association with surface conduction abnormalities in a large MMD1 population. Furthermore, we found no association between myocardial fibrosis by CMR-LGE and PPM implant which is a major clinical need for SCD prevention in MMD.

In our study the distribution of midwall myocardial fibrosis by LGE-CMR was predominantly in the septum (77%) followed by inferior and inferolateral regions (18%) – these findings are consistent with prior literature. In the study by Petri et al. there were 12 patients with LGE positivity, 50% exhibited fibrosis in the septum (anteroseptum and hinge points) and 33% in the inferior and inferolateral regions [9]. Similarly, Hermans and colleagues noted that 8 of the 10 patients with LGE positivity exhibited fibrosis in the septum and inferolateral wall [10]. In light of recent literature in the non-ischemic population demonstrating even a small extent of septal LGE being associated with increased risk of adverse cardiovascular events [30], the LGE extent and distribution in MMD1 patients raise concern for the arrhythmogenic potential of myocardial fibrosis in this population and further solidify the role of CMR in MMD1.

Our study suggests that myocardial fibrosis and conduction system abnormality may be two distinct phenomena occurring simultaneously in the myocardium of subjects affected by MMD1 and possibly carrying an interrelated but independent risk for adverse cardiovascular events. Recent studies seem to support this hypothesis, suggesting a possible relation between molecular mechanisms and surface conduction abnormalities [31, 32]. An analysis performed in subjects with MMD1 demonstrated a significant reduction in the expression of a microRNA precursor (miR-1) leading to deregulation of two important miR-1 targets: Connexion 43 and cardiac L-type calcium channel which encode gap-junction and the main calcium channels in the heart, respectively. Their misregulation may contribute to the conduction abnormalities observed in this population.

An additional aspect of our study is data on both replacement and interstitial fibrosis. In this cohort of MMD1 patients, we identified and quantified replacement fibrosis by LGE, and quantified interstitial fibrosis utilizing T1 mapping to obtain ECV values. Although there was a trend towards higher ECV values in patients with surface conduction abnormality, ECV was not statistically significantly different between the two groups. Further studies are needed to elucidate the nature of interstitial fibrosis in MMD1 [22].

Another important result of our study is the demonstration of high prevalence of myocardial fibrosis by CMR-LGE in subjects without surface conduction abnormality (43%). This finding has two relevant implications: i) myocardial fibrosis by CMR-LGE and surface conduction abnormality can occur independently in MMD and ii) additional studies are needed to know if detection of fibrosis afforded by CMR-LGE helps in predicting tachyarrhythmia risk in MMD that may not be captured by conduction abnormalities. The presence of myocardial fibrosis by CMR-LGE plays a key role in multiple cardiomyopathies as an important prognosticator for worse cardiovascular outcomes, particularly ventricular arrhythmias and sudden cardiac death [33–37]. In patients with MMD, myocardial fibrosis has been observed both in CMR and autopsy studies, giving evidence of a possible link between myocardial fibrosis and SCD independently of surface conduction abnormality [9–11]. Therefore, the high prevalence of myocardial fibrosis by CMR-LGE in the absence of surface conduction abnormality supports CMR’s complementary role for risk stratification in these patients. Furthermore, the presence of myocardial fibrosis by CMR could guide early initiation of cardioprotective therapies aimed to reduce the fibrotic burden and subsequent LV dysfunction as has been validated in other forms of neuromuscular disease [38].

Finally, similar to MMD1, MMD type 2 also manifests with cardiac involvement [39, 40]. Conduction abnormalities present in a similar pattern to MMD1, although they are less prevalent and appear to be more variable between affected individuals. CMR also demonstrates abnormalities in LGE and T1 signal in MMD2 individuals [14, 40]. Further larger studies are needed to establish the relationship between conduction and CMR abnormalities in MMD2.

### Limitations

This is a single center, retrospective study and the results should be confirmed in a large, multicenter, prospective study powered for both bradyarrhythmias as well as tachyarrhythmic SCD events.

It could be speculated that lack of correlation between myocardial fibrosis by CMR-LGE with surface conduction abnormality could be related to relatively early course of disease from the point of view of myocardial structural abnormality; however our findings rather support the opposite conclusion for the following reasons: i) there is a high prevalence of myocardial fibrosis which demonstrates that myocardial structural abnormality was frequent in this population ii) LV mass index was in the low normal range, which in line with previous studies, confirms evident muscle wasting typical for MMD1 iii) finally the length of MMD1 (median > 12 years in the whole population) excludes the possibility of an early stage of the disease where myocardial structural abnormality could have been absent or not detectable with CMR. Although we did not see trends towards significance with respect to myocardial fibrosis by CMR-LGE between our two cohorts, and our study is one of the largest CMR based studies in MMD1, our acceptance of the null hypothesis may relate to our sample size.

As the intent of our manuscript was to determine the association between myocardial fibrosis by CMR-LGE and surface conduction abnormality, we retrospectively included all consecutive patients with contrasted CMR exams and ECGs performed within 6 months. Our study spanned nearly 5 years during which two different MOLLI sequences were used at our institution, MOLLI 3(3)3(3)5 initially and then MOLLI 5(3)3. This is a limitation of our study.

ECV values were calculated from ROIs placed in the interventricular septum from the mid short axis view, hence they are not representative of the whole myocardium. However, according to recent literature and consensus documents, septal segments have proven to be the most reproducible as they are less frequently affected by off-resonance artifacts [21, 22]. Additionally, basal short axis T1 maps were not obtained at the time of the clinical scan, thus our data is only representative of the mid-short axis slice.

## Conclusion

Myocardial fibrosis by CMR-LGE in patients with MMD1 is highly prevalent but not related to surface conduction abnormality meeting criteria for PPM implantation according to current guidelines.

Our findings support the concept that MMD1 is characterized by a complex phenotype where conduction system dysfunction and myocardial fibrosis assessed by CMR-LGE are independent phenomena possibly with additive but independent prognostic significance.

High prevalence of myocardial fibrosis by CMR-LGE in the absence of surface conduction abnormalities warrants longer term follow-up to understand how CMR informs risk stratification in this disease.

## Additional file

Additional file 1: **Table S1.** T1 mapping in patients with and without conduction abnormality. **Table S2.** Patient characteristics according to CMR-LGE findings. **Table S3**. Electrocardiographic Characteristics According to CMR-LGE findings. **Table S4.** Full Univariate Logistic Regression Analysis of Variables Associated with Surface Conduction Abnormality. **Fig. S1**. Representative ECG and CMR findings in the study cohort including T1 mapping**.** Panels A shows an patient with abnormal ECG with prolonged PR interval and right bundle branch block, his corresponding native T1 map in panel B (utilizing MOLLI 3(3)3(3)5) and post-contrast T1 map in panel C yielding an ECV of 24%. Panel D demonstrates septal and inferolateral midwall fibrosis in the same patient. Panel E demonstrates a patient without conduction abnormality; his pre- and post-contrast T1 maps in panels F and G (utilizing MOLLI 5(3)3) yield an ECV of 23% and panel H demonstrates fibrosis in the inferior RV insertion site.

## Abbreviation

AV: Atrioventricular
bSSFP: Balanced steady-state free precession sequences
CMR: Cardiovascular magnetic resonance
DMPK: Dystrophy protein kinase gene
ECG: Electrocardiogram
ECV: Extracellular volume fraction
EP: Electrophysiological
IVCD: non-specific intraventricular conduction delay
LA: Left atrium/left atrial
LAFB: Left anterior fascicular block
LBBB: Left bundle branch block
LGE: Late gadolinium enhancement
LPFB: Left posterior fascicular block
LV: Left ventricle/left ventricular
LVEF: Left ventricular ejection fraction
LVH: Left ventricular hypertrophy
MIRS: Muscular Impairment Rating Scale
MMD1: Myotonic Muscular Dystrophy type 1
MOLLI: MOdified Look-Locker Inversion Recovery
NYHA: New York Heart Association
PPM: Permanent pacemaker
RBBB: Right bundle branch block
SCD: Sudden cardiac death
