# Supplementary material for: Myocardial fibrosis by late gadolinium enhancement cardiovascular magnetic resonance in myotonic muscular dystrophy type 1: highly prevalent but not associated with surface conduction abnormality
Source: J Cardiovasc Magn Reson. 2019 May 2;21:26. doi: 10.1186/s12968-019-0535-6 (PMC6498496; doi:10.1186/s12968-019-0535-6)
Supplement: Supplementary file 1 — Table S1. T1 mapping in patients with and without conduction abnormality. Table S2. Patient characteristics according to CMR-LGE findings. Table S3. Electrocardiographic Characteristics According to CMR-LGE findings. Table S4. Full Univariate Logistic Regression Analysis of Variables Associated with Surface Conduction Abnormality. Fig. S1. Representative ECG and CMR findings in the study cohort including T1 mapping. Panels A shows an patient with abnormal ECG with prolonged PR interval and right bundle branch block, his corresponding native T1 map in panel B (utilizing MOLLI 3(3)3(3)5) and post-contrast T1 map in panel C yielding an ECV of 24%. Panel D demonstrates septal and inferolateral midwall fibrosis in the same patient. Panel E demonstrates a patient without conduction abnormality; his pre- and post-contrast T1 maps in panels F and G (utilizing MOLLI 5(3)3) yield an ECV of 23% and panel H demonstrates fibrosis in the inferior RV insertion site. [file 12968_2019_535_MOESM1_ESM.docx]

**Supplemental Table 1. T1 mapping in patients with and without conduction abnormality.**

| **CMR data** | **Whole Cohort**  **(N=52)** | **Conduction Abnormality Positive**  **(N=31)** | **Conduction Abnormality Negative**  **(N=21)** | **P value** |
| --- | --- | --- | --- | --- |
| Native T1 myocardium, ms (N) |  |  |  |  |
| MOLLI 3(3)3(3)5 | 982 ± 48 (23) | 1003 ± 47 (13) | 954 ± 36 (10) | 0.012 |
| MOLLI 5(3)3 | 1013 ± 44 (29) | 1011 ± 52 (18) | 1017 ± 25 (11) | 0.740 |
| Post-contrast T1 myocardium, ms (N) |  |  |  |  |
| MOLLI 3(3)3(3)5 | 380 ± 40 (22) | 369 ± 41 (13) | 395 ±34 (9) | 0.133 |
| MOLLI 5(3)3 | 438 ± 50 (29) | 437 ± 55 (18) | 439 ± 42 (11) | 0.904 |
| ECV, %, both MOLLI sequences (N) | 25 ± 3 (51) | 26 ± 3 | 24 ± 3 | 0.050 |
| ECV by MOLLI sequence, % (N) |  |  |  |  |
| MOLLI 3(3)3(3)5 | 25 ± 3 (22) | 26 ± 4 (13) | 24 ± 2 (9) | 0.075 |
| MOLLI 5(3)3 | 25 ± 3 (29) | 26 ±3 (18) | 24±3 (11) | 0.332 |

**Notes**: Data are presented as mean ± SD or N (%). MOLLI: MOdified Look-Locker Inversion Recovery; ECV: extracellular volume.

**Supplemental Table 2. Patient characteristics according to CMR-LGE findings**

|  | **Whole Cohort**  **(N=52)** | **LGE**  **Positive**  **(N=22)** | **LGE**  **Negative**  **(N=30)** | **P value** |
| --- | --- | --- | --- | --- |
| **Demographic data** |  |  |  |  |
| Age, years | 41.2 ± 13.9 | 45.7 ± 14.1 | 37.9 ± 13.0 | 0.048* |
| Male gender, N (%) | 20 (38) | 10 (45) | 10 (33) | 0.403 |
| BMI, kg/m^2^ | 25.5 ± 5.8 | 25.9 ± 5.6 | 25.1 ± 5.9 | 0.617 |
| DM, N (%) | 5 (10) | 2 (9) | 3 (10) | 0.648 |
| HTN, N (%) | 3 (6) | 1 (4) | 2 (7) | 0.617 |
| Smoking, N (%) | 2 (4) | 1 (4) | 1 (3) | 0.999 |
| HLP, N (%) | 5 (10) | 3 (14) | 2 (7) | 0.639 |
| History of CAD, N (%) | 1 (2) | 0 (0) | 1 (3) | 0.999 |
| BB, N (%) | 8 (8) | 3 (14) | 1 (3) | 0.299 |
| ACE-i, N (%) | 3 (6) | 1 (4) | 2 (7) | 0.999 |
| ARB, N (%) | 1 (2) | 0 (0) | 1 (4) | 0.999 |
| MCRA, N (%) | 4 (8) | 1 (4) | 3 (10) | 0.629 |
| Statin, N (%) | 6 (12) | 4 (18) | 2 (7) | 0.382 |
|  |  |  |  |  |
| **Myotonic Muscular Dystrophy Characteristics** | | | | |
| Age of onset, years | 28.8 ± 15.5 | 34.4 ± 15.9 | 24.7 ± 14.0 | 0.027* |
| Disease length, years | 12.4 ± 12.7 | 11.3 ± 11.4 | 13.2 ± 13.8 | 0.593 |
| CTG Repeats | 500 (200-1163) | 580 (120-1381) | 450 (200-1115) | 0.939 |
| MIRS scale | 3 (3-4) | 3 (3-3) | 3 (3-4) | 0.756 |
|  |  |  |  |  |
| **Clinical data** | | | | |
| SBP, mmHg | 126 ± 18 | 131 ± 20 | 122 ± 16 | 0.128 |
| DBP, mmHg | 74 ± 10 | 76 ± 9 | 73 ± 10 | 0.268 |
| HR, bpm | 74 ± 14 | 74 ± 14 | 74 ± 15 | 0.942 |
| NYHA class | 2 (1-2) | 2 (1-2) | 1 (1-2) | 0.756 |
| Hematocrit, % | 42.1± 3.8 | 42.2 ± 4 | 42.0 ± 3.7 | 0.956 |

### Notes: Data are presented as mean ± SD, N (%), or median (interquartile range). *p < 0.05 considered significant. BMI: body mass index; DM: diabetes mellitus; HLP: hyperlipidemia; CAD: coronary artery disease; BB: beta-blockers; ACE-i: Angiotensin-converting enzyme inhibitors; ARB: Angiotensin receptor blockers; MCRA: aldosterone antagonists; MIRS: Muscular Impairment Rating Scale; SBP: systolic blood pressure; DBP: diastolic blood pressure; HR: heart rate; NYHA: New York Heart Association class

### **Supplemental** Table 3. Electrocardiographic Characteristics According to CMR-LGE findings

|  | | | | |
| --- | --- | --- | --- | --- |
| **Electrocardiology Data** | **Whole Cohort**  **(N=52)** | **LGE positive**  **(N=22)** | **LGE negative**  **(N=30)** | **P value** |
| Sinus bradycardia, N (%) | 19 (36) | 10 (45) | 9 (30) | 0.197 |
| PR, ms | 199 ± 47 | 205 ± 63 | 196 ± 32 | 0.531 |
| PR > 200 ms, N (%) | 28 (53) | 11 (50) | 17 (57) | 0.779 |
| QRS, ms | 103 ± 20 | 103 ± 17 | 104 ± 22 | 0.822 |
| QRS ≥ 120 ms, N (%) | 8 (15) | 4 (13) | 4 (18) | 0.708 |
| QT, ms | 404 ± 42 | 416 ± 39 | 396 ± 43 | 0.076 |
| QTc | 428 ± 32 | 431 ± 30 | 425 ± 35 | 0.500 |
| QTc > 450, N (%) | 12 (23) | 7 (23) | 5 (23) | 0.999 |
| Frontal QRS-T angle | 41 ± 41 | 47 ± 41 | 37 ± 41 | 0.399 |
| Absolute Cornell voltage, mm | 15 ± 8 | 15 ± 8 | 15 ± 9 | 0.914 |
| LVH-Cornell, N (%) | 13 (25) | 8 (27) | 5 (23) | 0.999 |
| LBBB, N (%) | 2 (4) | 1 (3) | 1 (4) | 0.999 |
| RBBB, N (%) | 6 (12) | 2 (9) | 4 (13) | 0.999 |
| IVCD, N (%) | 14 (27) | 3 (14) | 11 (36.7) | 0.060 |
| Fascicular block (LAFB, LPFB), N (%) | 17 (33) | 6 (27) | 11 (37) | 0.341 |
| Bifascicular block, N (%) | 6 (11.5) | 1 (4.5) | 5 (16.7) | 0.183 |
| AH interval, ms | 88 ± 21 | 82 ±15 | 97 ± 25 | 0.120 |
| HV interval, ms | 67 ± 15 | 64 ±17 | 71 ± 13 | 0.275 |
| **Surface Conduction Abnormality** | 31 (60) | 13 (59) | 18 (60) | 0.999 |

**Notes**: Data are presented as mean ± SD or N (%). LVH: left ventricular hypertrophy; LBBB: left bundle branch block; RBBB: right bundle branch block; IVCD non-specific intraventricular conduction delay; LAFB: left anterior fascicular block; AH: atrial-His interval; HV: His-ventricle interval.

### **Supplemental Table 4.** Full Univariate Logistic Regression Analysis of Variables Associated with Surface Conduction Abnormality.

| **Single Predictors of PMK implant** | | | |
| --- | --- | --- | --- |
| **Variables** | **Exp (B) or OR** | **95% CI** | **P value** |
| Age | 1.036 | 0.992-1.081 | 0.109 |
| Gender (Male) | 0.733 | 0.236-2.282 | 0.592 |
| Body mass index, kg/m2 | 1.025 | 0.929-1.131 | 0.624 |
| DM | 2.963 | 0.307-28.57 | 0.348 |
| HTN | 0.317 | 0.027-3.737 | 0.361 |
| Smoking |  |  | 0.999 |
| HLP | 2.963 | 0.307-28.57 | 0.348 |
| History of CAD |  |  | 0.999 |
| BB | 2.143 | 0.207-22.13 | 0.522 |
| ACE-i | 1.379 | 0.117-16.26 | 0.798 |
| ARB |  |  | 0.999 |
| MRA |  |  | 0.999 |
| Statin | 1.407 | 0.234-8.480 | 0.709 |
| Age of onset, years | 0.994 | 0.959-1.031 | 0.756 |
| Disease length, years | 1.071 | 1.003-1.144 | 0.040* |
| CTG Repeats | 0.999 | 0.999-1.001 | 0.259 |
| MIRS scale | 1.531 | 0.853-2.749 | 0.154 |
| SBP, mmHg | 0.996 | 0.966-1.027 | 0.792 |
| DBP, mmHg | 0.990 | 0.935-1.049 | 0.740 |
| HR, bpm | 0.995 | 0.957-1.035 | 0.799 |
| NYHA class | 0.838 | 0.349-2.011 | 0.692 |
| LVEF (%) | 0.981 | 0.895-1.075 | 0.681 |
| LVEDVI, ml/m^2 | 0.992 | 0.951-1.035 | 0.718 |
| LVESVI, ml/m^2 | 0.997 | 0.919-1.081 | 0.940 |
| LV mass index, g/m^2 | 1.017 | 0.954-1.085 | 0.600 |
| LAVI, ml/m^2 | 1.014 | 0.961-1.069 | 0.617 |
| LGE-CMR | 0.963 | 0.314-2.953 | 0.947 |
| LGE mass | 1.010 | 0.865-1.179 | 0.902 |
| LGE % | 0.998 | 0.884-1.127 | 0.973 |
| ECV, % | 3467 | 0.547-NP | 0.057 |
| Surface QRST angle | 1.005 | 0.991-1.020 | 0.459 |
| Cornell voltage, mm | 1.090 | 1.007-1.179 | 0.033* |

Notes: OR is computed for unit-change for continuous and ordinal predictors

**Supplemental Figure 1. Representative ECG and CMR findings in the study cohort including T1 mapping.**


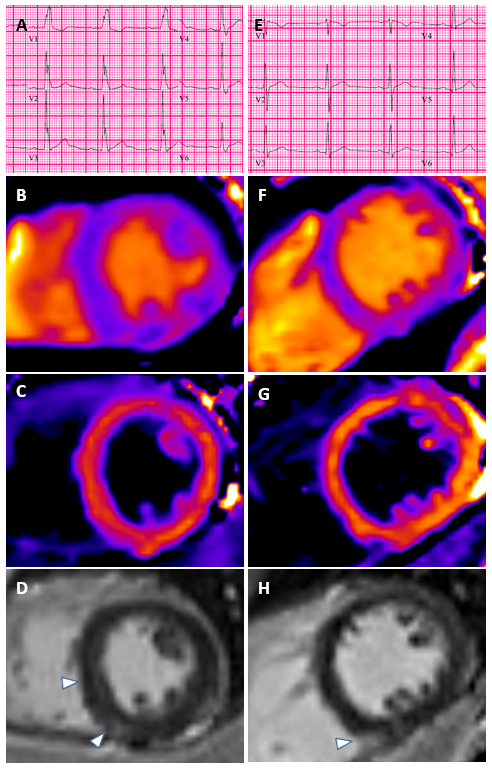


**Supplemental Figure 1 Legend:**

Panels A shows an patient with abnormal ECG with prolonged PR interval and right bundle branch block, his corresponding native T1 map in panel B (utilizing MOLLI 3(3)3(3)5) and post-contrast T1 map in panel C yielding an ECV of 24%. Panel D demonstrates septal and inferolateral midwall fibrosis in the same patient. Panel E demonstrates a patient without conduction abnormality; his pre- and post-contrast T1 maps in panels F and G (utilizing MOLLI 5(3)3) yield an ECV of 23% and panel H demonstrates fibrosis in the inferior RV insertion site.
